# Oleuropein aglycone induces autophagy *via* the AMPK/mTOR signalling pathway: a mechanistic insight

**DOI:** 10.18632/oncotarget.6119

**Published:** 2015-10-14

**Authors:** Stefania Rigacci, Caterina Miceli, Chiara Nediani, Andrea Berti, Roberta Cascella, Daniela Pantano, Pamela Nardiello, Ilaria Luccarini, Fiorella Casamenti, Massimo Stefani

**Affiliations:** ^1^ Department of Experimental and Clinical Biomedical Sciences, Division of Pharmacology and Toxicology, University of Florence, Florence, Italy; ^2^ Department of Neuroscience, Psychology, Drug Research and Child Health, Division of Pharmacology and Toxicology, University of Florence, Florence, Italy

**Keywords:** oleuropein aglycone, autophagy, neurodegeneration, AMPK, mTOR, Gerotarget

## Abstract

The healthy effects of plant polyphenols, some of which characterize the so-called Mediterranean diet, have been shown to arise from epigenetic and biological modifications resulting, among others, in autophagy stimulation. Our previous work highlighted the beneficial effects of oleuropein aglycone (OLE), the main polyphenol found in the extra virgin olive oil, against neurodegeneration both in cultured cells and in model organisms, focusing, in particular, autophagy activation. In this study we investigated more in depth the molecular and cellular mechanisms of autophagy induction by OLE using cultured neuroblastoma cells and an OLE-fed mouse model of amylod beta (Aβ) deposition. We found that OLE triggers autophagy in cultured cells through the Ca^2+^-CAMKKβ–AMPK axis. In particular, in these cells OLE induces a rapid release of Ca^2+^ from the SR stores which, in turn, activates CAMKKβ, with subsequent phosphorylation and activation of AMPK. The link between AMPK activation and mTOR inhibition was shown in the OLE-fed animal model in which we found that decreased phospho-mTOR immunoreactivity and phosphorylated mTOR substrate p70 S6K levels match enhanced phospho-AMPK levels, supporting the idea that autophagy activation by OLE proceeds through mTOR inhibition. Our results agree with those reported for other plant polyphenols, suggesting a shared molecular mechanism underlying the healthy effects of these substances against ageing, neurodegeneration, cancer, diabetes and other diseases implying autophagy dysfunction.

## INTRODUCTION

An increasing body of evidence points to a number of natural polyphenols as protective tools against cell sufferance and death in a wide number of human pathologies spanning from neurodegenerative to cardiovascular diseases, cancer and diabetes, including ageing. These molecules include compounds such as resveratrol, epigallocatechin-3-gallate (EGCG), curcumin, morin, quercetin and oleuropein aglycone (OLE) found in a number of foods of plant origin and believed to be at the basis of the claimed beneficial properties of these foods [1]. The wide interest raised around these molecules as potential tools to combat such a wide range of diseases or, at least, to reduce the severity of their symptoms and/or age of occurrence has prompted many groups to investigate various aspects of their physiological actions both at the cellular and molecular level and in animal models of a number of pathological conditions, particularly cancer, inflammatory, cardiovascular and neurodegenerative diseases [2,3]. A number of shared properties of these compounds includes their anti-oxidant, anti-inflammatory and anti-amyloidogenic power, and many benefits following their intake have been rationalised in the light of these recognised effects. However, more recently, other properties of these compounds arising from their interaction with cellular systems and potentially explaining their beneficial effects have been described. One of these is their ability to raise, in a number of different cell types, the autophagic response involved in protection against neurodegeneration, liver and vascular diseases [4-6].

Autophagy is a key process involved in cell homeostasis of proteins (proteostasis), lipids and organelles and contributes to clear materials of endogenous or exogenous origin. Together with the ubiquitin-proteasome system, autophagy participates to cellular proteostasis as one of the two main pathways of intracellular proteolysis, even though the former applies to the short-lived intracellular proteins, whereas the latter concerns proteins with longer half-lives. Autophagy encompasses the different routes used by cells to deliver cytoplasmic substrates to lysosomes through the formation of autophagosomes that subsequently fuse with lysosomes to form autophagolysosomes [7]. Usually, autophagy is triggered upon starvation, however it can be activated also by the presence of deposited materials as well as to degrade aged cellular organelles, principally mitochondria (mitophagy). Autophagy efficiency declines with age, with consequent accumulation of harmful protein aggregates and damaged mitochondria, which leads to increased ROS production [8]. The resulting increased cell stress favors inflammation and cell death [9].

Canonical autophagy, also known as macroautophagy, is a highly conserved process shared by all organisms which can be triggered in different ways and requires the participation of over 20 ATG proteins in yeast. The most straightforward path requires the inhibition of a cytoplasmic kinase receptor, the mammalian target of rapamycin (mTOR). mTOR forms two functionally distinct protein-mTOR complexes (mTORC1 and mTORC2) that are at the centre of an intricate, and still incompletely described, signalling pathway that is activated in response to a number of stress stimuli [10]. At the onset of the process, these complexes control the activation of the autophagy-initiating kinase (ULK1). mTORC1 inactivation in response to nutrient deprivation results in the activation of the ULK1 complex with ensuing induction of autophagy [7]. Autophagy senses, among others, the energy status of the cell and therefore responds to any cytosolic alteration of the AMP/ATP ratio which, in turn, signals to a number of kinases including liver kinase B1 (LKB1) and adenosine monophosphate-dependent protein kinase (AMPK). AMPK is activated upon phosphorylation by a number of upstream kinases including LKB1 and calmodulin-dependent kinase kinase β (CaMKKβ ), the latter in response to free Ca^2+^ increase, following the stimulation of the cAMP-inositol 1,4,5-triphosphate (IP3)/Akt and calpain-G-stimulatory protein α (Gsα) pathways [11]. In turn, activated AMPK promotes autophagy by phosphorylation and activation of ULK1 [12] and inhibition of mTORC1.

Autophagy abnormalities may contribute to many different pathophysiological conditions [13]; thus autophagy is increasingly considered as a promising target to treat a number of pathologies, particularly those associated with neurodegeneration [10] as well as ageing [14]. Actually, increased mTOR activity has been reported in cellular and animal models of Alzheimer's disease (AD) [15] as well as in Huntington animal models and disease [16, 17]; inducing autophagy can also be beneficial in amyotrophic lateral sclerosis [18].

Plant polyphenols are able to regulate autophagy in several ways. In particular, some polyphenols, such as resveratrol and EGCG, appear to promote the autophagic pathway by increasing cytosolic Ca^2+^ levels and activating AMPK by the CaMKKβ [4-6, 8]. Our previous data have highlighted the beneficial effects of OLE against protein/peptide aggregation *in vitro* [19, 20]. More importantly, our findings showed that TgCRND8 mice, a strain widely used as a model of amylod beta (Aβ) peptide deposition, fed with OLE displayed strongly improved performance in behavioural and cognitive tests; this effect was paralleled by reduced plaque load and plaque disassembly, in the affected brain areas, reduced inflammatory response, recovered dysfunctions of transgene-induced long-term potentiation (LTP) in the CA1 hippocampal area and reduced production of the pyro-Glu-Aβ 3-42 peptide, a recognised amyloid nucleator. These effects were, at least in part, accompanied and explained by epigenetic modifications [21] and, most remarkably, by a strong activation of autophagy [22, 23]. Autophagy stimulation by OLE agrees with the data previously reported for other plant polyphenols [24, 25], however, at variance with those, our data did not highlight any mechanistic explanation. To fill this gap and to expand the knowledge in the field not only in cultured cells but also in model animals, we investigated the molecular and cellular mechanisms of autophagy induction by OLE both *in vitro* in neuroblastoma SH-SY5Y cells and *in vivo* in TgCRND8 mice.

## RESULTS

### OLE induces a biphasic increase in AMPK phosphorylation at its regulatory Thr172

**Figure 1:**
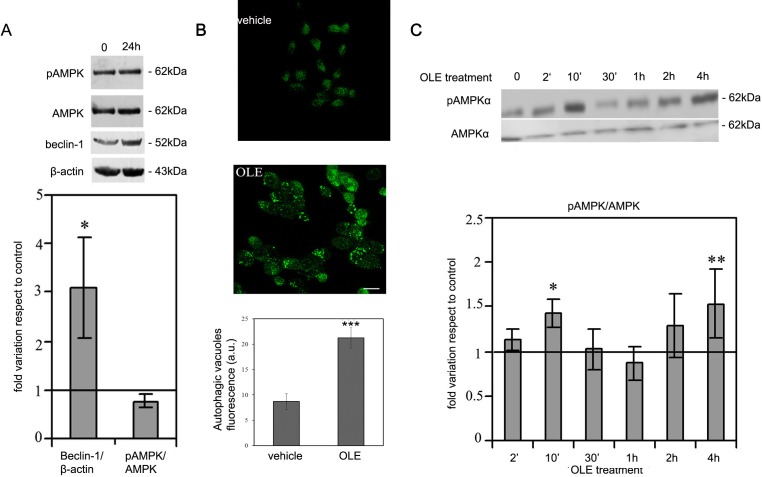
OLE induces autophagy and a biphasic increase in AMPK phosphorylation during short treatments SH-SY5Y cells were treated with OLE for 24 h, then **A.** (Upper panel) beclin-1 expression and AMPK phosphorylation were analysed by immunoblotting. β-actin was used to normalize beclin-1 expression. (Lower panel) Quantitative data. **B.** Treatment with OLE for 4 h determined an increase of autophagic vacuoles staining, detected by the Cyto ID-green Dye. Scale bar: 25 μm. **C.** (Upper panel) A biphasic increase in AMPK phosphorylation, detected by immunoblotting analysis, was evident in cells treated with OLE for increasing time periods. (Lower panel). Quantitative data. All data are Mean ± S.E.M. The asterisk (*) indicate a significant statistical difference with respect to vehicle-treated cells. *P < 0.05 **P < 0.01 *** *P* < 0.001. *n* = 3.

We previously showed that diet supplementation with OLE strongly ameliorates AD-associated symptoms in TgCRND8 mice, a model of Aβ deposition, in several ways, including induction of autophagy [21-23]; a similar behaviour was also shown in OLE-treated murine N2a neuroblastoma cells [23]. We therefore sought to elucidate the molecular mechanism underlying autophagy activation by investigating at which level OLE interfered with the autophagy cascade in SH-SY5Y human neuroblastoma cells. Previous data suggested that other polyphenols, such as resveratrol and EGCG, promote the autophagy flux by increasing the cytosolic Ca^2+^ levels with subsequent activation of AMPK by CaMKKβ [4-6]. Therefore, our primary aim was to assess if the molecular mechanism of autophagy induction in OLE-exposed SH-SY5Y cells was similar to that previously reported for other natural polyphenols. To do this, we initially exposed the cells to 50 μM OLE for 24 h, the conditions we previously reported to trigger autophagy in N2a cells [23] and then checked the cells for both Beclin-1 level (whose increase is an early marker of autophagy) and AMPK phosphorylation. However, no variation in the phosphorylation of the AMPK catalytic subunit at the regulatory Thr172 residue was observed at these conditions in spite of a significant increase in Beclin-1 expression (Figure 1A).

This negative result prompted us to explore whether an hypothetical OLE-mediated AMPK activation was an early event that disappeared after 24 h of cell treatment. In order to reduce the time frame of our treatments, at first we checked if autophagy was induced in SH-SY5Y cells after only 4 h of cell treatment with 50 μM OLE. At these conditions, autophagic vacuoles staining was evident suggesting that autophagy was indeed triggered even at this short time of treatment (Figure 1B). Accordingly, we analysed the phosphorylation level of AMPK within this time interval. We observed a biphasic significant increase of AMPK phosphorylation with respect to vehicle-treated cells after both 10′ and 4 h of OLE treatment (Figure 1C), supporting a close relation between autophagy induction and AMPK phosphorylation following cell exposure to OLE for reduced time periods.

### OLE induces a biphasic increase of the intracellular Ca^2+^levels

**Figure 2:**
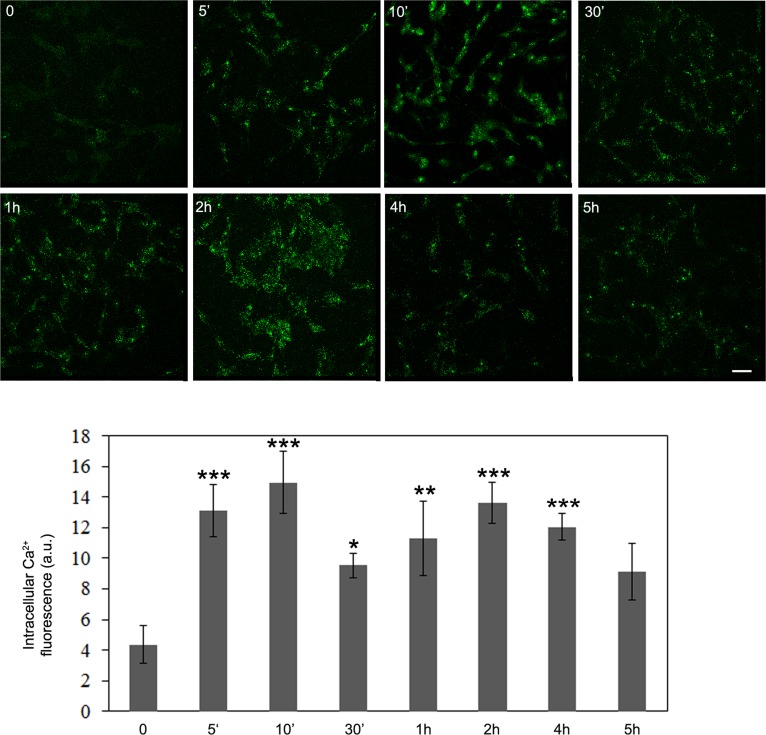
OLE induces a biphasic increase in intracellular Ca^2+^ levels during short treatments SH-SY5Y cells were treated with 50 μM OLE for different time periods. At the end of each treatment, the cells were washed with PBS and incubated for 30 min at 37°C with 10 μM Fluo-3AM as probe for detection of cytosolic calcium levels. Asterisks (*) indicate significant statistical differences with respect to time zero. *P < 0.05, **P < .0.01, *** *P* < 0.001. Scale bar: 25 μm *n* = 3.

It is well known that AMPK phosphorylation can result from the activation of the Ca^2+^-dependent Ser-Thr kinase CaMKKβ, a mechanism that accounts for autophagy induction by resveratrol and EGCG [4-6]. Therefore, we sought to explore whether, in our conditions, CaMKKβ was indeed involved even in the relation between cell treatment with OLE and the ensuing AMPK phosphorylation. To this purpose, we investigated the trend of the increase of intracellular free Ca^2+^ in OLE-treated cells to check whether it was associated to the biphasic character of AMPK phosphorylation. By using the fluorescent probe Fluo3-AM we found an early increase of the cytosolic Ca^2+^ after 5′-10′ of OLE treatment, with a subsequent decrease at 30′ (yet significantly above the basal level) followed by a late increase after 1-2 h, while after 5 h the Ca^2+^ level was no more significantly different from the basal level (Figure 2). These data suggested a temporal correlation between cytosolic Ca^2+^ increase and AMPK phosphorylation in OLE-treated cells.

**Figure 3:**
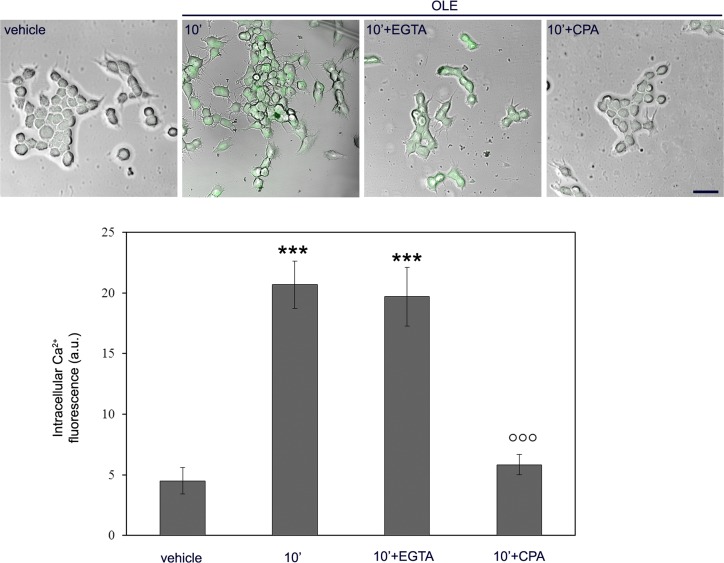
OLE determines the release of Ca^2+^ from the intracellular stores after 10′ of cell stimulation SH-SY5Y cells were pre-treated with CPA or EGTA before exposure to OLE for 10′. Cytosolic calcium levels were detected using the Fluo-3AM probe and the fluorescent signal was superimposed to phase-contrast images of the cells. Scale bar: 25 μm. The asterisks indicate significant statistical differences respect to vehicle treated cells, the circles indicate statistically significant differences with respect to OLE-treated cells. ***P < 0.001, °°° *P* < 0.001. *n* = 3.

Then, we focused at the early peak of Ca^2+^ increase and checked whether, in OLE-treated cells, the increased Ca^2+^ came from the extracellular medium or it was released from the intracellular stores. To do this, the cells, before treatment with OLE for 10 min, were incubated for 30 min with the cell-impermeant Ca^2+^ chelator EGTA or with CPA, an inhibitor of the endoplasmic reticulum (ER) Ca^2+^ pump which causes depletion of the ER stores. At these conditions, we found that only CPA abolished the OLE-induced Ca^2+^ increase, whereas cell treatment with EGTA did not induce any significant effect (Figure 3), suggesting that the free Ca^2+^ increase in OLE-treated cells resulted from ER release. We concluded that, in OLE-treated cells, AMPK phosphorylation paralleled Ca^2+^ increase coming from the ER, which suggested a pivotal role of the Ca^2+^-activated CaMKKβ.

### CaMKKβ activation is needed for OLE-autophagy induction

**Figure 4:**
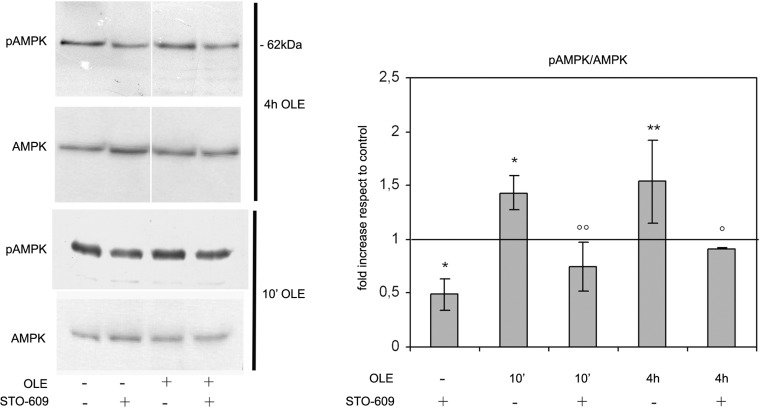
OLE-induced AMPK phosphorylation depends on CaMKKβ signalling SH-SY5Y cells were pre-treated with the CaMKKβ inhibitor STO-609 before adding OLE for 10′ or 4 h. (Left) Cellular extracts were analysed by immunoblotting. (Right) Quantitative data. The asterisks indicate a statistically significant difference respect to vehicle treated cells, the circles indicate statistically significant differences with respect to OLE-treated/STO-609-untreated cells. *P < 0.05, ** *P* < 0.01, ° *P* < 0.05, °°*P* < 0.01 *n* = 3.

To investigate whether CaMKKβ activation was indeed involved in the OLE-triggered AMPK activation, the cells were incubated with STO-609, a CAMKKβ inhibitor, prior to treatment with OLE for 10 min or 4 h. The subsequent western blotting analysis showed that, when CaMKKβ was inhibited, the level of AMPK phosphorylation was significantly lower than that measured both in the presence of OLE and in the absence of STO-609 and did not differed significantly from that found in OLE-untreated controls (Figure 4).

**Figure 5:**
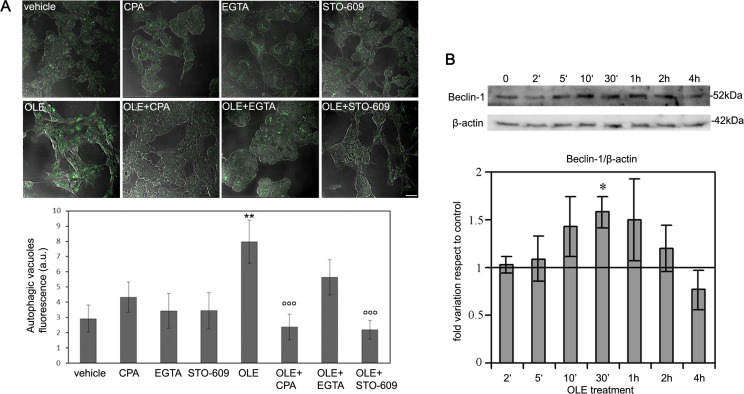
Short time cell treatment with OLE efficiently triggers the autophagic cascade SH-SY5Y cells were pre-treated with STO-609, CPA or EGTA before treatment with OLE for 10 min’. Then the cells were washed and incubated for 5 h in complete fresh medium. **A.** (Upper panel) Autophagy induction was measured by staining autophagic vacuoles with the Cyto ID green Dye. Scale bar: 30 μm. (Lower panel) Quantitative data. **B.** (Upper panel) Time course of beclin-1 expression during -short time treatments with OLE. β-actin was used to normalize beclin-1 expression. (Lower panel) Quantitative data. The asterisks (*) indicate statistically significant differences with respect to vehicle treated cells, the circles (°) indicate statistically significant differences with respect to OLE-treated cells. **P < 0.01, °°° *P* < 0.001 *n* = 3.

The above reported data suggest that cell treatment with OLE for 10 min induces a rise of free Ca^2+^ coming from the ER stores, which results in CaMKKβ activation and subsequent AMPK phosphorylation. Therefore, we investigated whether such a short-time treatment was sufficient to trigger autophagy and, if so, whether Ca^2+^ increase and CaMKKβ activation did play a role. So, the cells were pre-treated with EGTA, CPA or STO-609 for 30 min and then incubated with OLE for 10 min. Then the cells were washed and incubated for 5 h in complete fresh medium after which autophagy induction was measured. At these conditions, the cells displayed a significant increase in autophagic vacuoles staining (Figure 5A), suggesting that autophagy activation resulting from Ca^2+^ release from the ER in OLE-treated cells is an early event, and that longer times of incubation with OLE are not needed for autophagy induction. In fact, the latter was absent in cells pre-treated with CPA, confirming that Ca^2+^ release from the ER induced by a short acute (10 min) stimulus by OLE is essential for autophagy activation. We also confirmed the involvement of the Ca^2+^-activated CaMKKβ in such signalling cascade, since cell pre-treatment with STO-609 abolished OLE-induced increase of autophagic vacuoles staining. On the contrary, cell pre-treatment with EGTA did not inhibit autophagy, suggesting that the extracellular Ca^2+^ plays a minor role in the signalling cascade, at least in cells treated with OLE for short time periods.

The above findings are supported by the analysis of the time course of Beclin-1 expression that showed a slight, yet significant, transitory increase during the first minutes of OLE treatment, reaching a peak at 30 min (Figure 5B). Considering the short time of incubation with OLE, it is unlikely that such an increase stems from raised synthesis; rather it could arise from the Beclin-1 fraction complexed with Bcl-2/Bcl-xL in the cytoplasm. Actually, release of Beclin-1 from its complex with Bcl-2 is critical for inducing autophagy as free Beclin-1-VPS34 interaction is needed to initiate autophagosome formation [26]. A more consistent later increase in Beclin-1 expression had been already observed in cells exposed for 24 h to OLE (Figure 1). We conclude that cell exposure to OLE for a time as short as 10 min is sufficient to increase free cytosolic Ca^2+^ coming from the ER which, in turn, activates CaMKKβ with ensuing induction of AMPK phosphorylation and activation of the autophagic flux.

### AMPK activation is needed for autophagy induction by OLE

**Figure 6:**
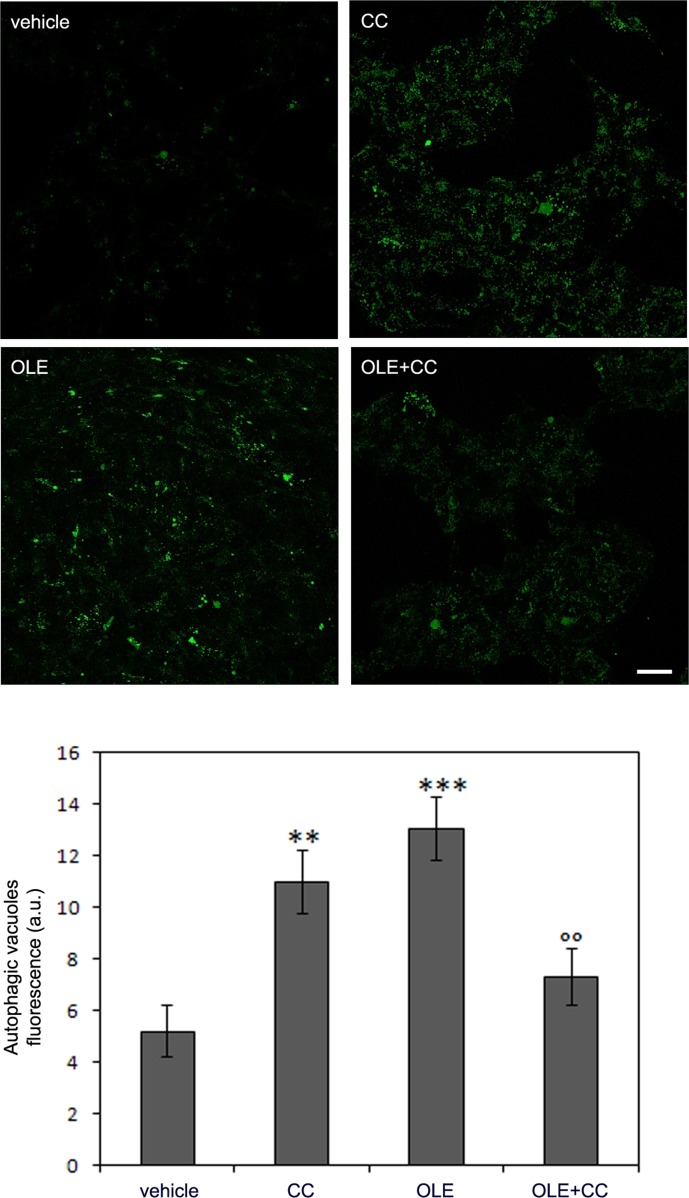
The AMPK inhibitor Compound C (CC) induces autophagy *per se* in SH-SY5Y and reduces OLE-induced autophagy The SH-SY5Y cells were treated with OLE in the presence or in the absence of CC. (Upper panel) Autophagy induction was measured by staining autophagic vacuoles with the Cyto ID green Dye. Scale bar: 25 μm. (Lower panel) Quantitative data. The asterisks (*) indicate a statistically significant difference respect to vehicle-treated cells, the circles (°) indicate a statistically significant difference with respect to OLE treated cells. **P < 0.01, ***P < 0.001, °° *P* < 0.01. *n* = 3.

**Figure 7:**
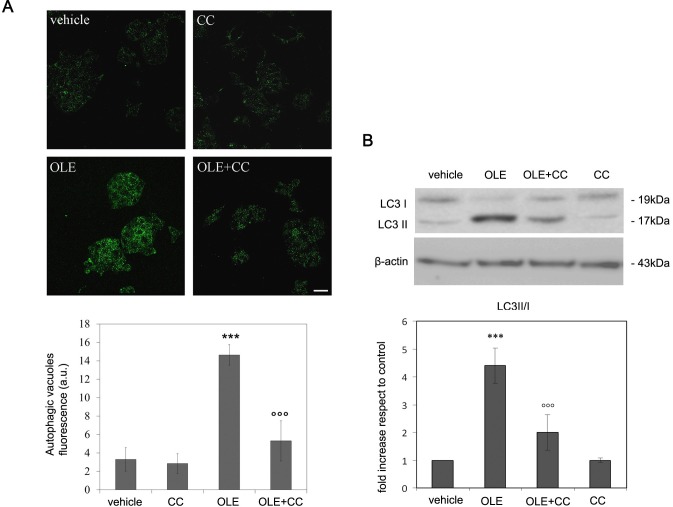
CC blocks autophagy induction by OLE in RIN-5F cells RIN-5F cells were pre-treated with CC before exposure to OLE for 4 h **A.** (Upper panel) Autophagic vacuole staining was detected by the Cyto ID-green Dye. Scale bar: 25 μm. (Lower panel) Quantitative data. **B.** (Upper panel) Cell extracts were analysed by immunoblotting with anti-LC3. β-actin was used to assess equal protein loading. (Lower panel) Quantitative data. The asterisks (*) indicate a statistically significant difference with respect to vehicle-treated cells. The circles (°) indicate a statistically significant difference with respect to OLE-treated cells. *** *P* < 0.001, °°°*P* < 0.001 *n* = 3.

Finally, we evaluated the correlation between AMPK activation and autophagy induction by OLE using compound C (CC), the only known AMPK inhibitor. We expected no autophagy induction in the presence of CC; however, we observed that autophagy was still activated, though to a reduced extent, at these conditions. To explain this effect, we checked whether CC induced basic autophagy in control cells; actually, we observed that CC is an autophagy activator *per se* (independently of any effect on AMPK activity) in this cell line (Figure 6), as it was previously reported for other cancer cells [27, 28]. Nevertheless, CC, even though did not completely abolish, significantly reduced OLE-autophagy activation, suggesting a strong prevalence of the pathway proceeding through AMPK even in this case. To confirm this hypothesis, we used a different, CC-insensitive, cell line chosen after testing a panel of different lines. We found an useful model in the rat pancreatic insulinoma RIN-5F cells, in which CC does not trigger autophagy. In these cells, OLE-treatment activated autophagy similarly to what observed in SH-SY5Y cells but this effect was significantly counteracted by cell pretreatment with CC, as shown both by autophagic vacuoles staining (Figure 7A) and by western blot analysis of LC-3 II (Figure 7B). These results fully confirmed, in a different cell line, that the activation of AMPK signaling plays a paramount role in OLE-induced autophagy, supporting the hypothesis that autophagy activation occurs mainly, if not exclusively, through the path OLE - Ca^2+^ increase - CaMKKβ activation - AMPK phosphorylation.

### OLE inhibits mTOR via activation of AMPK in the cortex of TgCRND8 mice

**Figure 8:**
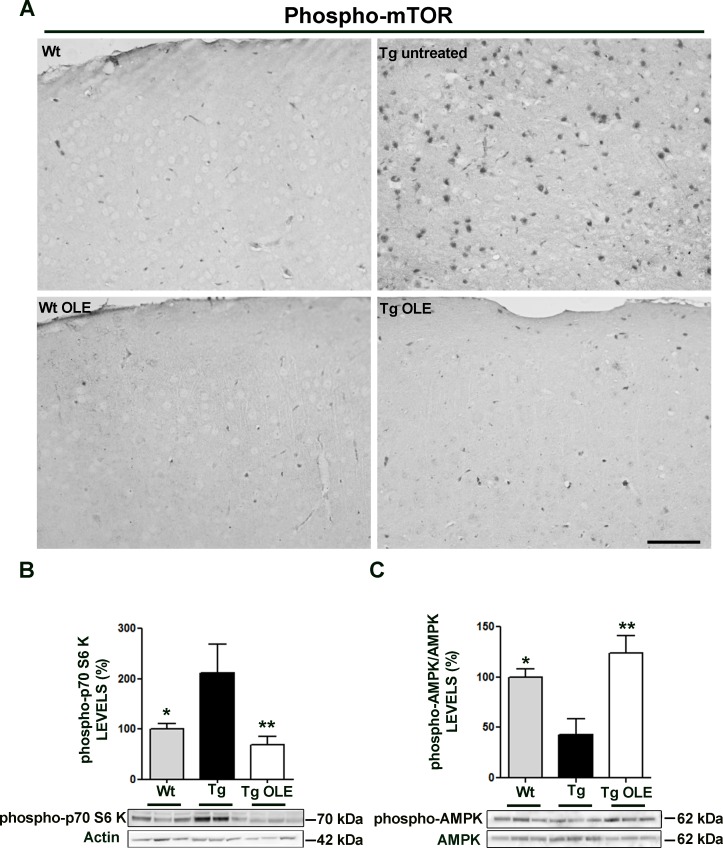
OLE restores to control levels the expression of phospho-mTOR, phospho-p70S6K and phospho-AMPK in the cortex of 6-12-month-old TgCRND8 mice **A.** Representative photomicrographs of phospho-mTOR immunoreactivity in the cortex of 12-month-old mice; note, the less intense phospho-mTOR staining in OLE-fed Tg mice, in untreated and OLE-fed wt mice compared with untreated Tg mice. Scale bar: 50 μm. **B.**, **C.** (Lower panel) Analysis of phospho-p70S6K and phospho-AMPK, detected by immunoblotting, respectively. (Upper panel) Quantitative data. The asterisks (*) indicate a statistically significant difference with respect to untreated Tg mice. *P < 0.05, **P < 0.01 (*n* = 5-6 mice/group).

To confirm and extend in tissue the proposed mechanism of autophagy activation by OLE in cultured cells, we used TgCRND8 mice, an *in vivo* model, of Aβ deposition which mimics some features of AD. We previously reported that OLE supplementation for 8 weeks to daily food of TgCRND8 mice induces an intense autophagic response and lysosomal activity in the cortex of animals of different ages (23). On the basis of these data, we explored the involvement of mTOR and AMPK in OLE-triggered autophagy in the cortical tissue of 6/12 month-old TgCRND8 mice fed with OLE. As expected, in the cortex of control, OLE-untreated, TgCRND8 mice we found enhanced phospho-mTOR immunoreactivity (Figure 8A) and increased levels of the phosphorylated mTOR substrate p70 S6K (Figure 8B); however, these modifications were markedly reduced in the cortex of OLE-fed TgCRND8 mice, where no significant differences with controls were detected. These data further support the idea that autophagy activation by OLE proceeds through mTOR inhibition. Next, we assessed whether the latter matched AMPK activation; we found enhanced levels of phospho-AMPK, and hence AMPK activation, in the cortex of OLE-fed TgCRND8 mice as compared to untreated animals (Figure 6C). Altogether, these data indicate that in the cortex of OLE-fed TgCRND8 mice the activation of autophagy likely occurs following AMPK activation, and that the latter matches mTOR inhibition. No age-related differences in both mTOR and AMPK data were detected in 6 month-old and in 12 month-old mice, thus the two age groups were pooled and reported as 6/12 months. Taken together, these results confirm those obtained with cultured cells and match those previously reported with a different neuroblastoma cell line [23], confirming that comparable molecular mechanisms of OLE-autophagy activation are present both in cultured cells and in tissue.

## DISCUSSION

A growing body of research has shown that polyphenols of plant origin have beneficial properties against ageing and a number of pathological conditions spanning from cancer, cardiovascular and neurodegenerative diseases, age-related metabolic deterioration and diseases including type 2 diabetes and the metabolic syndrome and neurodegenerative diseases. The presence of plant polyphenols at substantial levels in a number of foods that characterize the Mediterranean and the Asian diets, has been recognized as a leading explanation of the benefits for health of these alimentary regimens [1, 29]. Plant polyphenols possess a number of properties that can contribute to explain their effects on health, the most recognized being their antioxidant, anti-inflammatory and anti-aggregation power. However, these properties, although of importance, cannot explain by themselves the remarkable effects of these molecules that appear not generic but, rather, very specific, even though shared. In fact, they are able to impact on cell viability by interfering with a number of basic processes including transcriptional regulation through epigenetic effects and autophagy [8, 21, 23].

Recent research has sought to decipher the polyphenol-cell biology relations by investigating some of these substances that are present in foods of large use including red wine, green tea, extra virgin olive oil and the turmeric. The data reported in recent years have shown that specific polyphenols such as resveratrol, EGCG and curcumin are able to elicit the autophagic response thus improving such housekeeping tool that results in increased cell longevity and favors the clearance of misfolded/aggregated peptides and proteins in several human amyloid diseases [8].

It is widely known that autophagy activation can result from different pathways including the phospho-inositol-(IP3) signalling, the Ca^2+^-calpains-G_s_*_α_* pathways [30], in addition to the Ca^2+^ -CaMKKβ (LKB1)-AMPK-mTOR pathway. The molecular basis of autophagy activation by a number of polyphenols have been investigated; they include the pivotal role of AMPK, a heterotrimeric Ser/Thr kinase, that is activated by several upstream kinases, including LKB1 and CaMKKβ, and, in turn, negatively controls mTOR, a potent inhibitor of autophagy [31-33]. CaMKKβ is activated by free Ca^2+^, whose cytosolic levels appear important to initiate the autophagy flux. Ca^2+^ increase seems to be the main upstream biochemical modification induced by several polyphenols as well as a primary cause that eventually leads to the activation of autophagy. However, at present, it cannot be concluded that this is a basic effect shared by plant polyphenols known to be protective against ageing and disease, nor whether different polyphenols trigger different autophagy pathways.

Recently, we have shown that, in the TgCRND8 mice, OLE administration with the food results in reduction of Aβ peptide production and neuroinflammation; more remarkably, it induces other significant effects, restoring LTP, reducing the number and compactness of the plaques, activating the microglia and, most importantly, promoting an intense autophagic response [21, 23]. However, nothing was known at the molecular level about the mechanism of the latter effect. In this study we tried to fill the gap by investigating some molecular mechanisms underlying autophagy activation by OLE both *in vitro* in neuronal cells and *in vivo* in TgCRND8 mice. In the latter, the activation of AMPK which, in turn, negatively modulates mTOR, could underlie the previously reported OLE-induced intense autophagic and lysosomal response in the brain cortex of OLE-fed mice [23].

Here, we found that OLE induces autophagy in cultured neuroblastoma cells by a mechanism substantially in accordance with those previously reported for other polyphenols, confirming that in cells treated with polyphenols the Ca^2+^/CaMKKβ/AMPK/mTOR axis is actually at work. We found that the Ca^2+^ rise in OLE-treated cells was the result of Ca^2+^ exit from the intracellular stores rather than of its entry from the external medium. This implies that OLE penetrates inside the cell though the plasmamembrane without damaging it. We did not checked the presence of OLE inside the cells, however, this would not be surprising, considering that the molecule is hydrophobic. Moreover, such a possibility is supported by a recent report where it has clearly been shown that compounds from olive leaves, including OLE, are found inside treated cells in a breast-cancer cell line [34]. We have previously reported that a time-dependent decrease of phosphorylation of the mTOR substrate p70 S6K occurs in N2a cells, a different neuroblastoma cell line, treated with OLE at the same concentration used in this study [23], confirming that autophagy activation by OLE proceeds through AMPK activation and mTOR inhibition. Of interest, in the same study, AMPK activation was evident at 3-6 h of OLE treatment, a time similar to that at which, in this study, we found the highest peak of AMPK phosphorylation. Taken together, these data support the hypothesis that autophagy activation by OLE proceeds via the AMPK/mTOR signalling pathway. That a panel of coherent, yet not completely matching, results were found with different cultured cells does not weakens the take-home message of this study; rather it strengthens it, confirming that similar molecular mechanisms underlie OLE-induced activation of autophagy in different cell types as well as in mouse tissue.

The above considerations open intriguing consequences. For example, it has been repeatedly reported that some polyphenols, including OLE, are able to induce epigenetic modifications, in terms of histone acetylation and DNA methylation, particularly in cancer cells [35] and that, in the brain of OLE-fed TgCRND8 mice, modified expression levels of key proteins including glutaminyl cyclase, Beclin-1, LC-3 II and histone deacetylase-2 were found [21]. In such a scenario the transcriptor factor EB (TFEB), a transcriptional regulator of lysosomal biogenesis that controls the expression of central autophagy genes [4-6], could perform an important role; in fact, it appears to be inhibited upon phosphorylation by mTORC and activated upon dephosphorylation by calcineurin, a phosphatase that has recently been shown to be activated by lysosomal Ca^2+^ [36]. Hence, autophagy appears to be regulated by Ca^2+^ signals coming from different cell stores that affect independently but, possibly, synergistically, the autophagic flux. In this context, the Ca^2+^/CaMKKβ/AMPK/mTOR axis could synergize with calcineurin maintaining TFEB in a de-phosphorylated, and hence activated, form for longer time periods. An aspect that merits further investigation.

Another aspect of potential interest is represented by the positive control exerted by insulin and several growth factors on mTORC that should result in reduced ability to trigger autophagy and, possibly, reduced synergy to the calcineurin effects. Impaired insulin sensitivity, with increased insulin incretion, is a common phenomenon in aged people that can affect autophagy efficiency [37, 38]. Accordingly, type 2 diabetes could find a right place in such a scenario. Actually, it has recently been shown that autophagy activation through positive control of transcription of key autophagy genes is decreased under conditions of insulin resistance [39].

In conclusion, our study provides evidence that the mechanism of autophagy activation by polyphenols resides on quite general effects of these substances on cell regulatory mechanisms underlying the control of autophagy. Our data add further knowledge to the molecular basis of OLE-induced autophagy that confirms and extends our previous studies carried out both *in vivo* and with a different cell line. When added to the available literature, our findings provide strong support to the idea that plant polyphenols are indeed useful to treat ageing and age-related diseases where autophagy dysfunction plays an important role such as cancer, neurodegeneration and type 2 diabetes thus reinforcing the theoretical basis for their use to combat these conditions.

## MATERIALS AND METHODS

### Oleuropein deglycosylation

Oleuropein (Extrasynthase) deglycosylation was performed as previously described [19]. Oleuropein aglycone was dissolved to 100 mM in DMSO to obtain a stock solution that was stored at −20°C protected from light. Dilutions in aqueous buffers were made immediately before use.

### Cell cultures

Human SH-SY5Y neuroblastoma cells and rat RIN-5F insulinoma cells were from American Type Culture Collection (ATCC). SH-SY5Y cells were cultured in DMEM F-12/HAM (1:1) supplemented with 10% fetal calf serum (FCS, Sigma-Aldrich, Steinheim, Germany), glutamine and antibiotics. RIN-5F cells were cultured in RPMI medium supplemented with 10% FCS, 2.25 g/L glucose, 10 mM HEPES, 1.0 mM, sodium pyruvate, glutamine and antibiotics. Both cell lines were maintained in a 5.0% CO_2_ humidified atmosphere at 37°C.

### Analysis of autophagic vacuoles

The Cyto-ID^®^ Autophagy Detection Kit (Enzo Life Sciences) which uses a novel dye that selectively labels autophagic vacuoles in living cells was used to monitor autophagy induction by fluorescence microscopy, according to the manufacturer instructions. The cells were plated for 24 h in 24-well plates containing coverslips and then treated with 50 μM OLE for different time periods. In some experiments the cells were pre-treated for 30 min with 13.3 μM STO-609 (a CaMKKβ inhibitor), 4.0 mM EGTA (an impermeant Ca^2+^ chelator), 10 μM compound C (CC, an AMPK inhibitor) or for 1.0 h with 40 μM cyclopiazonic acid (CPA, an ER Ca^2+^-ATPase blocker (all inhibitors were from Sigma-Aldrich), before OLE administration. After incubation with OLE, the cells were washed and incubated for further 4.0 h in complete fresh medium. Then, the medium was removed and the cells were washed twice with 100 μl of PBS and then with 100 μl of 1 × Assay-Buffer provided with Dectection Kit containing 10% FCS. After washing, the cells were incubated for 30 min at 37°C with 100 μl of Dual detection reagent (prepared by diluting Cyto-ID Green Detection Reagent 330 times in a mixture of 1× Assay Buffer plus FCS), protected from light. Finally, the cells were washed three times with the same solution (1 × Assay Buffer-FCS 10%) and the coverslips were placed on microscope slides. The stained cells were analyzed by using a confocal Leica TCS SP5 scanning microscope (Mannheim, Germany) equipped with an argon laser source for fluorescence measurements at 488 nm and a Leica Plan Apo 63 × oil immersion objective. A series of optical sections (1024 × 1024 pixels) 1.0 μm in thickness was taken through the cell depth for each examined sample. The confocal microscope was set at optimal acquisition conditions, e.g., pinhole diameters, detector gain and laser powers. Settings were maintained constant for each analysis. Phase-contrast images were also acquired using the DIC channel. To quantify the green fluorescent signal, 10-22 cells were analyzed in each experiment using ImageJ software (NIH, Bethesda, MD).

### Western blotting

In parallel with autophagic vacuoles analysis, OLE-treated cells were analyzed by western blotting. To this purpose, the cells were lysed directly in Laemmli buffer (60 mM Tris-HCl pH 6.8, 2% (w/v) SDS, 10 mM EDTA, 10% (w/v) glycerol). Before immunoblotting, protein concentration was determined with a BCA detection kit (Pierce, USA) and adjusted to equal concentrations across different samples. The samples were added with β-mercaptoethanol and bromophenol blue, boiled for 10 min, clarified at 10000 × g for 10 min, run on 12% SDS-PAGE and transferred to PVDF membranes (Amersham Bioscience, UK). After blocking with 5.0% (w/v) BSA in 0.1% (v/v) PBS-Tween-20 or directly with methanol, the membranes were incubated overnight at 4°C with specific primary antibodies: rabbit polyclonal anti-beclin1 antibody (1:2000, Abcam), rabbit polyclonal anti-LC3II A/B antibody (1:1000, Cell Signaling), rabbit monoclonal anti-phospho-AMPKα (Thr172) and anti-AMPKα antibodies (1:1000, Cell Signaling). Mouse monoclonal anti-β−actin antibody (1:1000, Santa Cruz Biotechnology Inc.) was used for protein load normalization. The day after, the blots were incubated for 1.0 h with specific secondary antibodies (1:10000, goat anti-rabbit antibody and goat anti-mouse antibody, Molecular Probes, Life Technologies); the immunoreactive bands were detected with the Immobilon Western Chemiluminescent HRP substrate (Millipore) or SuperSignal West Pico chemiluminescent substrate (Thermo Fisher Scientific) and quantified by densitometric analysis using a ChemiDoc system and the Quantity One software (Bio-Rad Laboratories, Italy). Statistical analysis of the bands was performed on results from at least three independent experiments, using ANOVA and Bonferroni's post-test.

### Measurement of intracellular Ca^2+^ levels

The cytosolic levels of free Ca^2+^ were measured by using the Fluo-3-acetoxymethyl ester (Fluo-3AM) probe (Molecular Probes) whose fluorescence largely increases in response to Ca^2+^ binding. Subconfluent SH-SY5Y cells grown on glass coverslips were treated with 50 μM OLE for different time periods. At the end of each treatment, the cells were washed with PBS and incubated for 30 min at 37°C with 10 μM Fluo-3AM plus Pluronic F-127 (20% in DMSO) at a 1:1 (v/v) ratio (the final DMSO concentration used to dissolve the probe was 0.04%). Then the cells were washed again and fixed in 2.0 % buffered paraformaldehyde for 20 min. Cell fluorescence was analyzed by confocal microscopy, quantified and statistically analyzed as described for autophagic vacuoles analysis.

### Animal experiments

Transgenic (Tg) hemizygous CRND8 mice harboring a double-mutant gene of APP695 [40] and wild type (wt) control littermates were used following the ECC (DL 116/92, Directive 86/609/EEC) and National guidelines for animal care. The protocol was approved by the Committee on the Ethics of Animal Experiments of the Italian Ministry of Health (Permit Number: 283/2012-B). Tg and wt mice of 4 and 10 months of age at the beginning of treatment were used, *n* = 6 group/genotype, equally divided for sex. The animals were treated for 8 weeks with a modified low-fat (5.0%) AIN-76A diet (10 g/day per mouse) supplemented with OLE (50 mg/kg of diet) (OLE-fed mice) or not (untreated mice) as previously reported [21, 23]. Tissue processing, immunohistochemistry and western blotting were also as previously reported [21, 23]. In western blotting, 1:1000 rabbit anti-phospho-p70 S6 Kinase (Thr389), anti-phospho-AMPK (Thr172) and anti-AMPK (Cell Signaling, MA, USA) antibodies were used; anti-actin rabbit antibody (Sigma Aldrich, MO, USA) was diluted 1:4000. In immunohistochemistry experiments, a rabbit anti-phospho-mTOR (S2448) polyclonal antibody (Abcam, Cambridge, UK) was used at 1:100 dilution.

### Statistical analysis

One-way ANOVA, plus Bonferroni's post-hoc test, was used to analyse Western blotting experiments. Statistical analyses were carried out with GraphPad Prism version 5.0 for Windows (San Diego, CA, USA) and the statistical significance was defined as *p* < 0.05. Data are reported as mean values ± standard error of the mean (S.E.M).
